# Dose-Response Tendon-Specific Markers Induction by Growth Differentiation Factor-5 in Human Bone Marrow and Umbilical Cord Mesenchymal Stem Cells

**DOI:** 10.3390/ijms21165905

**Published:** 2020-08-17

**Authors:** Maria Camilla Ciardulli, Luigi Marino, Erwin Pavel Lamparelli, Maurizio Guida, Nicholas Robert Forsyth, Carmine Selleri, Giovanna Della Porta, Nicola Maffulli

**Affiliations:** 1 Department of Medicine, Surgery and Dentistry “Scuola Medica Salernitana”, University of Salerno, Via S. Allende, 1, 84084 Baronissi (SA), Italy; mciardulli@unisa.it (M.C.C.); lmarino@unisa.it (L.M.); elamparelli@unisa.it (E.P.L.); cselleri@unisa.it (C.S.); n.maffulli@qmul.ac.uk (N.M.); 2 Department of Neuroscience and Reproductive Science and Dentistry, University of Naples “Federico II”, Via Pansini, 5, 80131 Naples, Italy; maurizio.guida@unina.it; 3 Guy Hilton Research Centre, School of Pharmacy and Bioengineering, Keele University, Stoke-on-Trent ST4 7QB, UK; n.r.forsyth@keele.ac.uk; 4 Mile End Hospital, Centre for Sports and Exercise Medicine, Queen Mary University of London, Barts and the London School of Medicine and Dentistry, 275 Bancroft Road, London E1 4DG, UK

**Keywords:** Wharton’s Jelly human umbilical cord mesenchymal stem cells (hWJ-MSCs), Growth Differentiation Factor-5, human bone marrow Mesenchymal Stem Cells (hBM-MSCs), tenogenic commitment, gene expression, immunofluorescence assay

## Abstract

Mesenchymal stem cells derived from human bone marrow (hBM-MSCs) are utilized in tendon tissue-engineering protocols while extra-embryonic cord-derived, including from Wharton’s Jelly (hWJ-MSCs), are emerging as useful alternatives. To explore the tenogenic responsiveness of hBM-MSCs and hWJ-MSCs to human Growth Differentiation Factor 5 (hGDF-5) we supplemented each at doses of 1, 10, and 100 ng/mL of hGDF-5 and determined proliferation, morphology and time-dependent expression of tenogenic markers. We evaluated the expression of collagen types 1 (COL1A1) and 3 (COL3A1), Decorin (DCN), Scleraxis-A (SCX-A), Tenascin-C (TNC) and Tenomodulin (TNMD) noting the earliest and largest increase with 100 ng/mL. With 100 ng/mL, hBM-MSCs showed up-regulation of SCX-A (1.7-fold) at Day 1, TNC (1.3-fold) and TNMD (12-fold) at Day 8. hWJ-MSCs, at the same dose, showed up-regulation of COL1A1 (3-fold), DCN (2.7-fold), SCX-A (3.8-fold) and TNC (2.3-fold) after three days of culture. hWJ-MSCs also showed larger proliferation rate and marked aggregation into a tubular-shaped system at Day 7 (with 100 ng/mL of hGDF-5). Simultaneous to this, we explored the expression of pro-inflammatory (IL-6, TNF, IL-12A, IL-1β) and anti-inflammatory (IL-10, TGF-β1) cytokines across for both cell types. hBM-MSCs exhibited a better balance of pro-inflammatory and anti-inflammatory cytokines up-regulating IL-1β (11-fold) and IL-10 (10-fold) at Day 8; hWJ-MSCs, had a slight expression of IL-12A (1.5-fold), but a greater up-regulation of IL-10 (2.5-fold). Type 1 collagen and tenomodulin proteins, detected by immunofluorescence, confirming the greater protein expression when 100 ng/mL were supplemented. In the same conditions, both cell types showed specific alignment and shape modification with a length/width ratio increase, suggesting their response in activating tenogenic commitment events, and they both potential use in 3D in vitro tissue-engineering protocols.

## 1. Introduction

Tendon injuries affect the elderly, active workers, and athletes, resulting in marked economic healthcare and societal burden [1,2]. Clinical manifestation itself is the result of a long-term interplay of inflammatory and failed healing response changes [3]. The management of tendon injuries is challenging, given their limited healing capability, and propensity for a failed healing response [4,5]. Anti-inflammatory drugs are frequently used, but they may actually hinder recovery [6], while in clinical practice, tissue grafts or prostheses are used for severe injuries [7,8], and cell therapies are proposed for future application [9,10,11]. Since the present therapeutic modalities are only partially effective, a variety of regenerative medicine options are now of great interest. They aim to induce tendon healing and regeneration, focusing on the role of mesenchymal stem cells therapy and their derivatives [12,13].

Multipotent Mesenchymal Stem Cells (MSCs) are described as a promising therapeutic tool to manage tendon conditions in clinical applications [14]. Both in vivo and in vitro studies evidenced that MSCs can contribute to accelerate and improve the quality of tendon healing [15,16]. The use of MSCs, in combination with specific growth factors (GFs) is proposed as an innovative treatment for tendon healing and regeneration [17,18]. Human bone marrow MSCs (hBM-MSCs) have been extensively characterized [19]. hBM-MSCs are multipotent cells able to differentiate into various types of mesenchymal cell phenotypes, including osteoblasts, chondroblasts, myoblasts and tenocytes under specific conditions in vitro [20,21,22]. Their application in ligament and tendon reconstruction strategies is promising.

Given the huge gap of knowledge regarding the biology of tendon healing and the sequence of events and biomolecules involved, a diversity of growth factors are used for tendon research [2,23]. Among these, GDF family members, and more precisely, Growth Differentiation Factor 5 (GDF-5), have been used successfully to drive tenogenic differentiation. GDF-5 belongs to the TGF-β superfamily, and it is highly expressed in mesenchymal condensations during skeleton development. GDF-5 is also called cartilage-derived morphogenetic protein-1 (CDMP-1) and bone-derived morphogenetic protein-14 (BMP-14). BMPs are deeply involved in the development of endochondral bones [24] and joints [25], inducing the expression of type 1 collagen in connective tissue [26], with a consequential important role in tendon healing [27]. Indeed, GDF-5 induces the expression of genes linked to the neo-tendon phenotype [28,29,30], and its administration seemed to improve the outcome of tendon repair [31,32]. However, little is known about the effect of GDF-5 on BM-MSCs transcriptional regulation and differentiation [22]. Park et al., reported that GDF-5 supplemented (100 ng/mL) culture media enhanced extracellular matrix (ECM) and tenogenic marker gene expression in adipose-derived mesenchymal stem cells (ADMSCs) over 12 days [33]. GDF-5 also appeared to induce tenogenic differentiation of human BM-MSCs when used at a concentration of 100 ng/mL as reflected by significant increases in total collagen expression and tenogenic marker gene expression (Scleraxis, Tenascin-C and type 1 collagen at Day 7) [30]. Further, BMP-14 (50–100 ng/mL) significantly increased tendon marker expression (Scleraxis and Tenomodulin) at mRNA and protein level potentially via the Sirt1-JNK/Smad1-PPARγ signaling pathway and enhanced by TGF-β3 and VEGF. The association of BMP-14 with TGF-β3 and VEGF enhanced the tenogenic differentiation of BM-MSCs [22,34]. Pathway analyses on hBM-MSCs treated with GDF-5 highlighted that the potential molecular pathways involved in GDF-5 induced tenogenic differentiation included cytoskeleton reorganization, cell adhesion, and extracellular matrix signaling [30]. Further investigation demonstrated apparent cytoskeleton reorganization, suggesting an important event in tenogenic differentiation [35].

hBM-MSCs have been applied within 3D in vitro models of tendon healing and regeneration [36,37,38]. However, given the limited number of hBM-MSCs available for autologous use, donor site morbidity, and their limited proliferative capacity, it is important to identify alternative MSCs sources for clinical use and application in tissue-engineered protocols of tendon regeneration.

An alternative tissue source of MSCs is the connective tissue (Wharton’s Jelly) of the human umbilical cord (hWJ-MSCs) [39,40]. MSCs derived from extra-embryonic tissue, including hWJ-MSCs, share several characteristics with adult MSCs, but also retain some features of developmentally immature stem cell populations, i.e., broad germ layer-spanning differentiation potential, but do not induce teratomas formation and have a potential application in regenerative medicine that is not impeded by ethical or legal issues [41]. Moreover, hWJ-MSCs display high proliferation rates, wide multipotency, hypoimmunogenicity, and unlike hBM-MSCs, require a painless collection procedure and have faster self-renewal properties, all important features in cell-based therapies [41,42,43]. hWJ-MSCs have been studied for application in neurological disorders [44], kidney injury [45], lung injury [46], orthopedic injury [47], liver injury [48], and cancer therapy [49] this far. Mesenchymal stromal cells isolated from Wharton’s Jelly have been induced to form neurons, myocytes, bone, cartilage and adipose cells [40,50,51,52,53], but, except few recent studies in vivo [54,55], very little is known about their tenogenic commitment.

The repair process in injured tendons consists of three overlapping phases reported as the inflammatory, proliferative and remodeling stages [56]. Several cell types are involved in tendon healing. Pro-inflammatory (M1) and anti-inflammatory (M2) macrophages directly orchestrate tendon remodeling and by secreting cytokines and growth factors, activate the epithelial-to-mesenchymal transition (EMT) signaling cascades in the epithelial cells that surround tendon tissue, providing a source of mesenchymal cells able to repair the injured tissue [56]. Indeed, preclinical and clinical studies have demonstrated the anti-inflammatory and immunomodulatory potential of MSCs, even though the mechanisms behind the MSC-based immunomodulation remain a challenge. For example, MSCs can have different immunomodulatory effects, even on the same types of immune cells, depending on the disease status. However, both hBM-MSCs and hWJ-MSCs display immunomodulatory properties and produce large amounts of cytokines and growth factors, connected to the differentiation processes [41,57,58]. A correlation between GDF-5 and cytokine expression has been noted in human annulus cells in in vitro disc degeneration models where high levels of two pro-inflammatory cytokines (IL-1β and TNF-α) leads to a significant down-regulation of GDF-5 [59].

To date, there is no single growth factor (or a cocktail of growth factors) and protocols known to efficiently induce tenogenic differentiation of stem cells. Given this challenge, determining stem cell responses to specific dosages of GDF-5 treatment is a fundamental step in addressing tendon regeneration strategies, tissue-engineering models, and protocols. With the aim of exploring the possible use of hWJ-MSCs, instead of the gold standard hBM-MSCs, in tissue-engineering protocols for tendon healing and repair, here, we report the effect of a range of human GDF-5 (hGDF-5) concentrations on tendon and cytokine gene expression on hWJ-MSCs. hBM-MSCs are included to enable direct comparison with the research standard. Markers, such as type 1 collagen (COL1A1), Decorin (DCN), Scleraxis-A (SCX-A), Tenascin-C (TNC) and Tenomodulin (TNMD), and both pro-inflammatory (IL-6, TNF, IL-12A, IL-1β) and anti-inflammatory (IL-10, TGF-β1) cytokines, were monitored by Real-Time Polymerase Chain Reaction (RT-PCR). Type 3 collagen (COL3A1) was chosen as a negative marker because it is basally expressed by stem cells. Morphometric analysis with cells alignment, shape and length/width ratio coupled with type 1 collagen and tenomodulin proteins detection by quantitative immunofluorescence completed the proposed study.

## 2. Results

**Figure 1:**
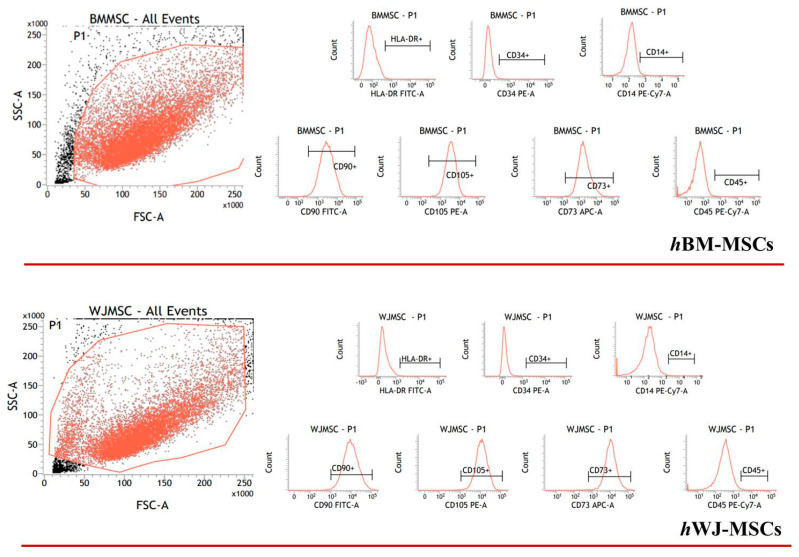
Flow cytometry events illustrate human bone marrow mesenchymal stem cells (hBM-MSCs) and human Wharton’s Jelly mesenchymal stem cells (hWJ-MSCs) characterization. The panel shows the representative flow cytometry event of forward scatter (FSC) vs. side scatter (SSC).

**Figure 2:**
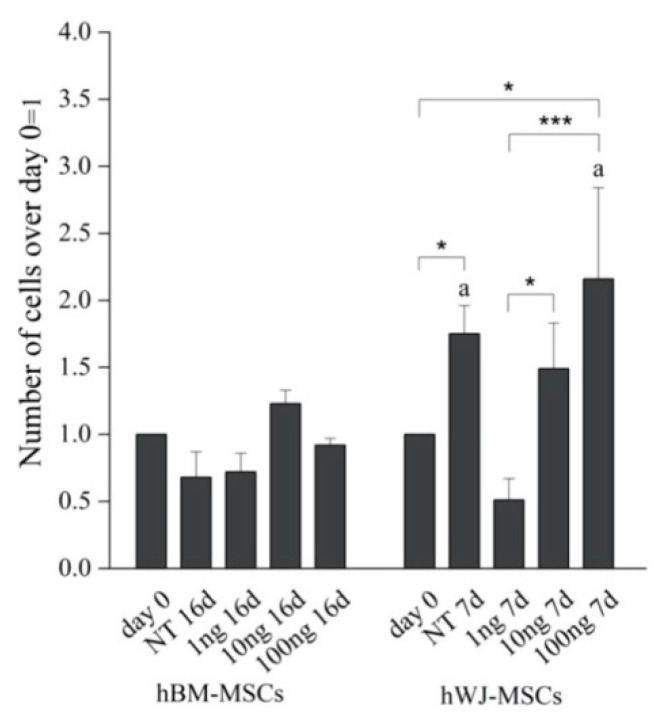
Proliferation rate of hBM-MSCs and hWJ-MSCs with the human Growth Differentiation Factor 5 (hGDF-5) dose-dependent effect. The proliferation rate was calculated over different culture time points and for both untreated cells and hGDF-5 treated cells. Statistically significant differences are shown as * = *p* ≤ 0.05; *** = *p* ≤ 0.005; a = *p* ≤ 0.05 compared to hBM-MSCs (NT 7d hWJ-MSCs vs. NT 16d hBM-MSCs; 100 ng 7 d hWJ-MSCs vs. 100 ng 16 d hBM-MSCs); *n* = 3. (NT = untreated cells; 1 ng = 1 ng/mL of hGDF-5; 10 ng = 10 ng/mL of hGDF-5; 100 ng = 100 ng/mL of hGDF-5; d = days).

Detailed flow cytometry characterization of both hBM-MSCs and hWJ-MSCs is reported in Table S1 (Supplementary Materials) and data acquisition profiles illustrated in Figure 1. All samples were positive for CD90, CD105, CD73 and negative for CD14, CD34, CD45, HLA-DR in accordance with previously published data [60]. The proliferation of both cell types was determined during the experimental time course both with and without hGDF-5 supplementation. No statistically significant differences in proliferative potential were noted for hBM-MSCs (at Day 16 for untreated cells -NT- it looks like cells number decreased though) for all the hGDF-5 concentration explored; while hWJ-MSCs displayed a generally higher proliferation rate (Figure 2) with respect the hBM-MSCs (see NT at Day 7) that was also positively affected by the growth factor concentrations.

**Figure 3:**
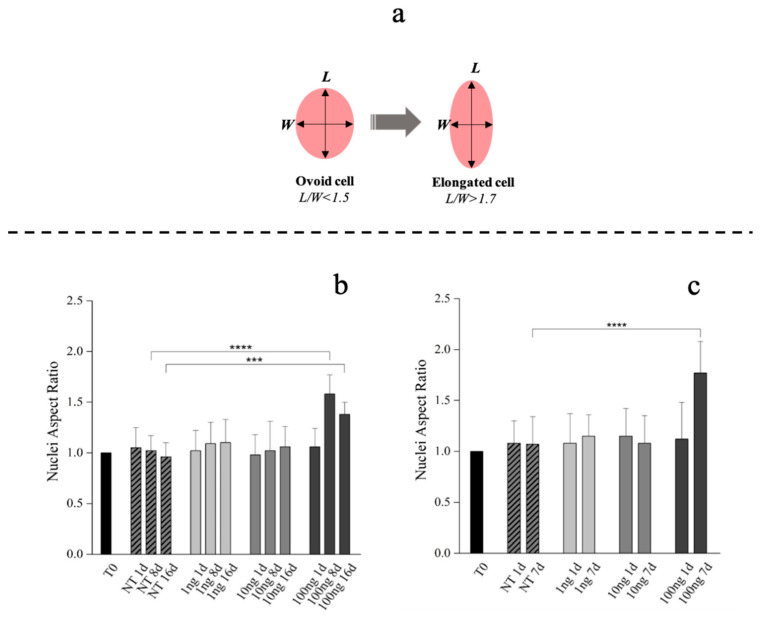
Morphometric analysis of hBM-MSCs and hWJ-MSCs with the hGDF-5 dose-dependent effect. Shape modification analysis illustrated the hGDF-5 concentration-dependent effect on cells (**a**). Nuclei aspect ratio vs. treated cells are reported for hBM-MSCs (**b**) and hWJ-MSCs (**c**); data on untreated cells are also reported for comparison purpose. Statistically significant differences are shown as *** = *p* ≤ 0.005; **** = *p* ≤ 0.001. (T0 = day 0; NT = untreated cells; 1 ng = 1 ng/mL of hGDF-5; 10 ng = 10 ng/mL of hGDF-5; 100 ng = 100 ng/mL of hGDF-5; d = days).

Compared to tenoblasts, whose nucleus is ovoid (nuclei length/width ratio <1.5), the overall tenocytes shape and their nucleus appears elongated with a nuclei length/width ratio >1.7 (Figure 3a) [61,62]. Therefore, hBM-MSCs phenotype commitment was evaluated by nuclear aspect ratio evolution (length/width: L/W) determined at Day 1, 8 and 16. hBM-MSCs also showed a specific alignment and shape modification (tenoblast-like) with an L/W ratio ≥1.5 when 100 ng/mL of hGDF-5 were supplemented at Day 8 and at Day 16 (Figure 3b and Figure S1 in Supplementary Materials). Extending our examination of hGDF-5 induction of tenogenic gene expression into hWJ-MSCs, we noted that cells displayed an aligned phenotype with a tenocyte-like shape, coupled to the L/W ratio value > 1.7, at Day 7 with 100 ng/mL hGDF-5 supplementation (Figure 3c and Figure S1 in Supplementary Materials).

**Figure 4:**
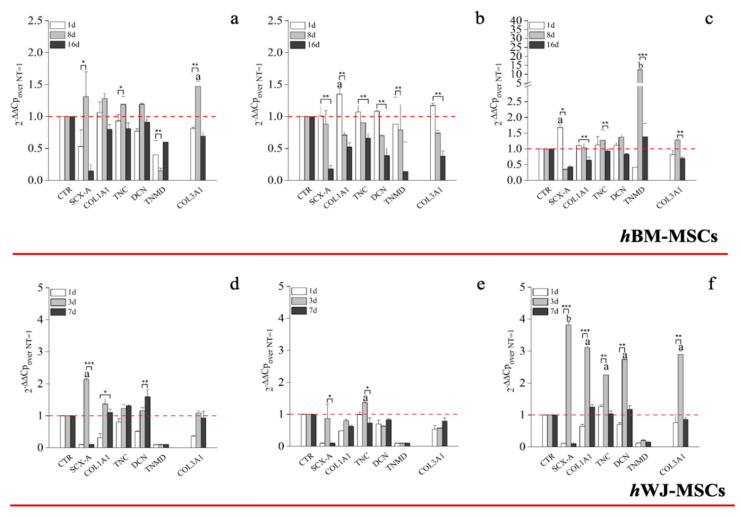
Real-Time Polymerase Chain Reaction (RT-PCR) on the expression of tenogenic markers by hBM-MSCs and hWJ-MSCs with the hGDF-5 dose-dependent effect. The mRNA levels of tenogenic markers (Type 3 collagen: COL3A1, type 1 collagen: COL1A1, Decorin: DCN, Scleraxis-A: SCX-A, Tenomodulin: TNMD, Tenascin-C: TNC) were monitored; three different concentration of hGDF-5 were tested: (**a**,**d**) 1 ng/mL, (**b**,**e**) 10 ng/mL and (**c**,**f**) 100 ng/mL. Untreated cells for matched time-points selected were used for control purposes. Statistically significant differences are shown as * = *p* ≤ 0.05, ** = *p* ≤ 0.01, *** = *p* ≤ 0.005; a = *p* ≤ 0.05, b = *p* ≤ 0.01 compared to NT.

Transcriptional analysis of hBM-MSCs supplemented with hGDF-5 (1 ng/mL) revealed up-regulation of SCX-A (1.3-fold), COL1A1 (1.3-fold), COL3A1 (1.5-fold), DCN (1.2-fold) and TNC (1.2-fold) after eight days of culture. Up-regulation was transient in nature and had returned to baseline levels or less by Day 16 (Figure 4a). An hGDF-5 concentration of 10 ng/mL resulted in a similar transcriptional up-regulation pattern as before, but that peaked at Day 1 and decreased thereafter (Figure 4b). With 100 ng/mL hGDF-5 supplementation, SCX-A was overexpressed at Day 1 with an up-regulation of 1.7-fold and at Day 8 TNMD (12-fold), DCN (1.4-fold), TNC (1.3-fold), COL1A1 (1.3-fold), and COL3A1 (1.2-fold) were observed (Figure 4c). All transcripts were down-regulated thereafter at Day 16.

Looking at RT-qPCR data concerning the expression of tenogenic markers by hWJ-MSCs, 1 ng/mL was associated with up-regulation of SCX-A (2-fold) and COL1A1 (1.3-fold) at Day 3 and DCN (1.5-fold) at Day 7 only (Figure 4d). Similarly, 10 ng/mL had little effect on tenogenic gene expression with the exception of TNC, which increased 1.3-fold at Day 3 (Figure 4e). In contrast to above, and similar to hBM-MSCs, 100 ng/mL hGDF-5 induced significant up-regulation of SCX-A (3.8-fold), COL1A1 (3-fold), TNC (2.3-fold), DCN (2.7-fold), and COL3A1 (2.9-fold) at Day 3 (Figure 4f).

**Figure 5:**
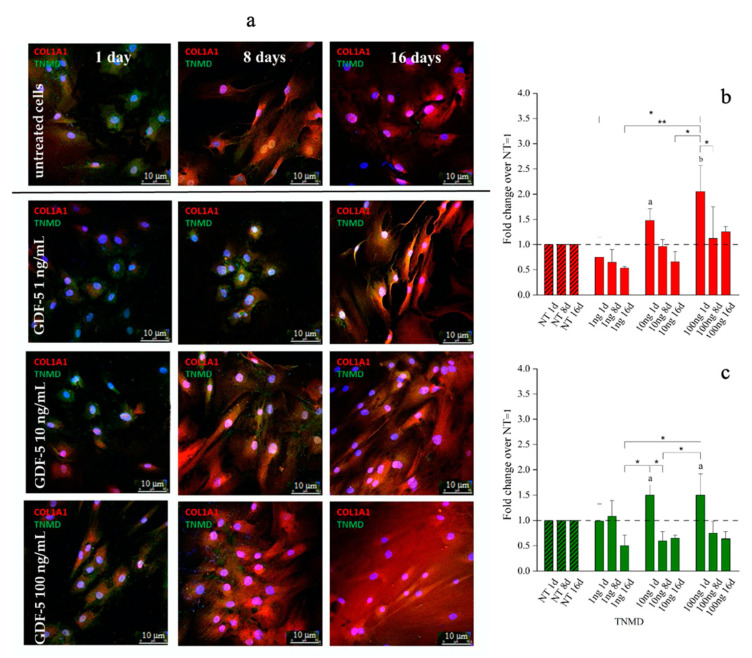
Immunofluorescence (IF) images and quantitative immunofluorescence (q-IF) data illustrating the effect of different hGDF-5 dose on hBM-MSCs. The panel (**a**) shows type 1 collagen (red staining) and tenomodulin (green staining) proteins up to 16 days of culture. Quantitative data on type 1 collagen (**b**) and tenomodulin (**c**) proteins were also reported; untreated cells (NT) are illustrated for comparison purpose. Statistically significant differences are shown as * = *p* ≤ 0.05; ** = *p* ≤ 0.01; a = *p* ≤ 0.05, b = *p* ≤ 0.01 compared to NT. (T0 = day 0; NT = untreated cells; 1ng = 1 ng/mL of hGDF-5; 10 ng = 10 ng/mL of hGDF-5; 100 ng = 100 ng/mL of hGDF-5; d = days).

**Figure 6:**
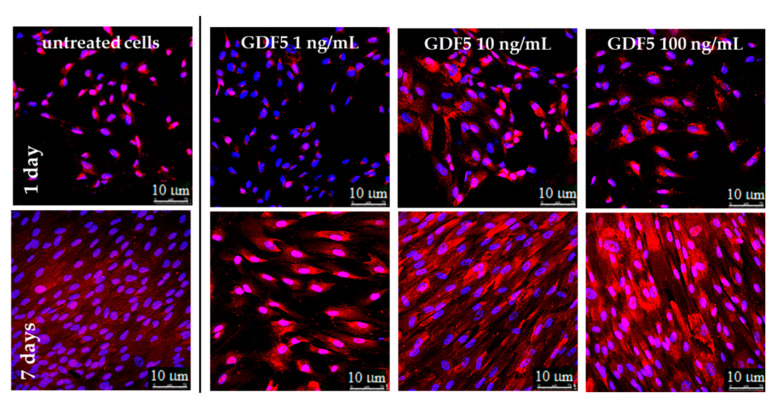
IF images illustrating the type 1 collagen expression at different hGDF-5 dose on hWJ-MSCs. Protein was stained in red and monitored up to seven days of culture.

**Figure 7:**
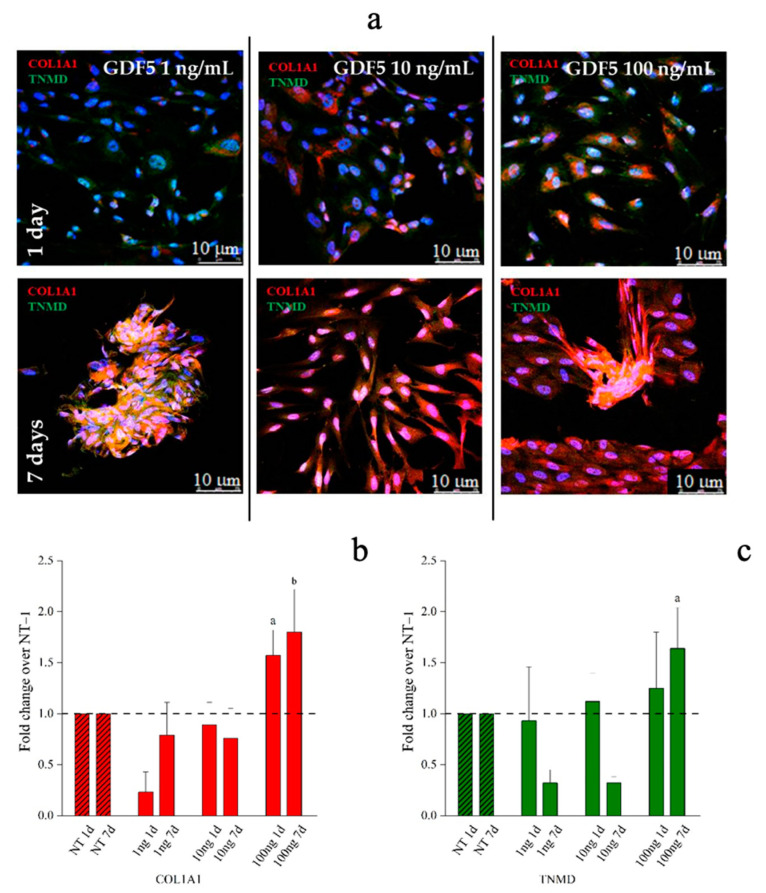
Immunofluorescence (IF) images and quantitative immunofluorescence (q-IF) data illustrating the effect of different hGDF-5 dose on hWJ-MSCs. The panel (**a**) shows type 1 collagen (red staining) and tenomodulin (green staining) proteins up to seven days of culture. Quantitative data on type 1 collagen (**b**) and tenomodulin (**c**) proteins were also reported; untreated cells (NT) are illustrated for comparison purpose. Statistically significant differences are shown as a = *p* ≤ 0.05, b = *p* ≤ 0.01 compared to NT. (T0 = day 0; NT = untreated cells; 1ng = 1 ng/mL of hGDF-5; 10 ng = 10 ng/mL of hGDF-5; 100 ng = 100 ng/mL of hGDF-5; d = days).

Type 1 collagen and tenomodulin protein expression were investigated by quantitative immunofluorescence assay across both hBM-MSCs and hWJ-MSCs populations. Representative images and their quantification are shown in Figure 5, Figure 6 and Figure 7, respectively. The protein expression by hBM-MSCs and hWJ-MSCs was similar even if the time points explored were necessarily different, due to the proliferation rate.

With regard to hBM-MSCs, 1 ng/mL of hGDF-5 was associated with no up-regulation of either protein. Type 1 collagen expression increased by 1.5-fold and 2-fold after treatment with 10 and 100 ng/mL of hGDF-5, respectively, at Day 1 (Figure 5b), and was accompanied by tenomodulin increase (1.5-fold) after treatment with 10 and 100 ng/mL of hGDF-5 at Day 1 (Figure 5c) (see also Figure S2 in Supplementary Materials). No cells aggregates were detected along the culture.

Similar to hBM-MSCs, untreated hWJ-MSCs showed an increase in basal type 1 collagen over time; however, the protein was only largely overexpressed when 100 ng/mL of hGDF-5 were supplemented. Moreover, these cells achieved an extremely ordinate alignment towards a specific direction and showed an elongation of their shape (Figure 6).

Aggregation into 3D spindle-like structures were observed when cells were treated with 1 ng/mL of hGDF-5 while tubular-like 3D structures were observed when 100 ng/mL of growth factor was used after seven days of treatment. This tubular structure was already reported by Barboni et al., and is considered an early organization of cellular 3D structure [63]. Tenomodulin was present within these spindle-shaped and tubular-shaped aggregates (Figure 7a and Figure S3 in Supplementary Materials). Quantification of immunofluorescence in hWJ-MSCs images indicated that the type 1 collagen signal was significantly increased by 1.5-fold and 2.0-fold after treatment with 100 ng/mL of hGDF-5 at Day 1 and Day 7, respectively (Figure 7b). TNMD staining showed a significant increase of 1.5-fold with 100 ng/mL of hGDF-5 at Day 7 (Figure 7c).

**Figure 8:**
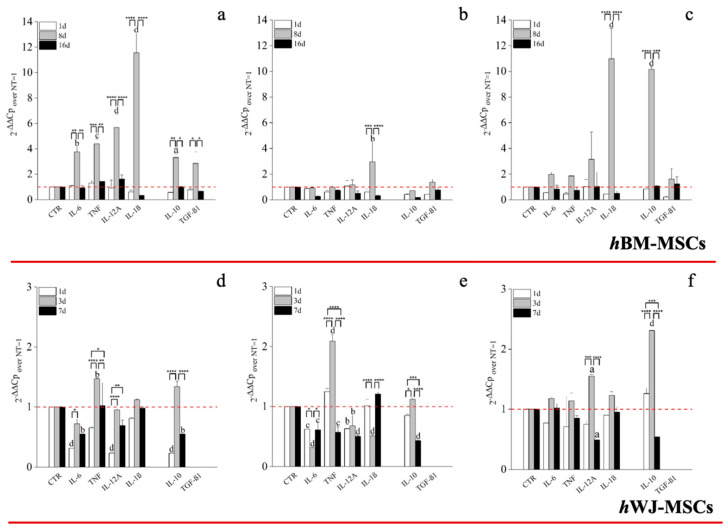
RT-PCR on the expression of cytokine by hBM-MSCs and hWJ-MSCs with the hGDF-5 concentration-dependent effect. The mRNA levels of both pro-inflammatory (IL-6, TNF, IL-12A and IL-1β) and anti-inflammatory (IL-10 and TGF-β1) cytokines were monitored at hGDF-5 concentrations of 1 ng/mL (**a**,**d**), 10 ng/mL (**b**,**e**) and 100 ng/mL (**c**,**f**). Untreated cells for matched time-points selected were used for control purposes. Statistically significant differences are shown as * = *p* ≤ 0.05, ** = *p* ≤ 0.01, *** = *p* ≤ 0.005, **** = *p* ≤ 0.001; a = *p* ≤ 0.05, b = *p* ≤ 0.01, c = ≤ 0.005, d = *p* ≤ 0.001 compared to NT.

A key correlator with tendon repair and MSCs function is immunological responsiveness and modulation. An hGDF-5 supplementation of 1 ng/mL with hBM-MSCs up-regulated both pro-inflammatory (IL-6: 4-fold; TNF: 4-fold; IL-12A: 6-fold and IL-1β: 11.5-fold) and anti-inflammatory (IL-10: 3-fold and TGFβ1: 2.5-fold) cytokines, at Day 8, but not thereafter (Figure 8a). Conversely, with 10 ng/mL up-regulation of IL-1β (3-fold), only, was noted at Day 8. (Figure 8b). The highest dose of hGDF-5, 100 ng/mL, was accompanied by significant up-regulation of IL-1β (11-fold) and IL-10 (10-fold) at Day 8, only (Figure 8c).

With regard to hWJ-MSCs, evaluation of pro- and anti-inflammatory cytokine expression following on from hGDF-5 supplementation revealed up-regulation of TNF (~1.5-fold) at Day 3 with 1 ng/mL (Figure 8d). With 10 ng/mL hGDF-5 supplementation, we again noted up-regulation of TNF (2-fold) at Day 3 (Figure 8e). Finally, with 100 ng/mL hGDF5 supplementation, we noted significant up-regulation of IL-12A (1.5-fold), and again, IL-10 (2.5-fold) at Day 3 (Figure 8f).

## 3. Discussion

We hypothesized that hMSCs from human bone marrow (hBM-MSCs) supplemented with hGDF-5 would result in up-regulation of genes and proteins consistent with tenogenic differentiation, and consequently, synthesize and secrete these into the extracellular environment. We further hypothesized a correlation between hMSCs differentiation and their immunomodulatory activity, through cytokines expression. We utilized hMSCs, isolated from the umbilical cord (hWJ-MSCs), to determine their comparability and behavior in the same conditions (three different concentrations of hGDF-5) and their possible in vitro use in tendon tissue-engineering. Different doses of hGDF-5 (1 ng/mL, 10 ng/mL and 100 ng/mL) were selected to explore a dose-response effect with regard to tenogenic commitment and cytokine profile behaviors. As reported in the introduction, the dosage range explored is generally recognized to have a biological action in vitro within tenogenic commitment studies [29,30,33]; higher concentrations of 1000 ng/mL seemed to induce cells apoptosis, and were not discussed in the present study (data not shown).

It is well recognized in the literature that MSCs derived from adult sources have a very low proliferation potential [64]. Therefore, we were able to explore hBM-MSCs behavior for up to 16 days, acquiring further data with respect to previous studies reported in the literature which described the characterization of the same cells against a maximum of 10 days [30]. hWJ-MSCs showed several similarities, but also some differences. First, given their faster population doubling levels [41,42,43], culture longer than seven days was not possible, even though several and considerably lower seed densities were explored.

Our data are in strong agreement with previous literature in confirming the dose of 100 ng/mL as optimal for cells commitment toward a tenogenic phenotype. This observation was confirmed by morphometric analyses, that evidenced nuclei elongation with an increase of nuclei L/W ratio. hBM-MSCs showed specific tenoblast-like phenotype with an L/W ratio ≥1.5 when 100 ng/mL of hGDF-5 were supplemented at Day 8 and at Day 16. Instead, a tenocyte-like shape, coupled to the L/W ratio >1.7 was achieved by hWJ-MSCs after only seven days of treatment when 100 ng/mL of hGDF-5 was supplemented. In the case of hWJ-MSCs, we have not acquired images for intermediate time points, but, looking at the results of the morphometric analysis in Figure 3c, cells supplemented with 100 ng/mL of GDF-5 were significantly more elongated than untreated cells at Day 7, and it was not observed in the control—therefore, it is cannot be considered an effect of confluency. Moreover, we noticed specific tenogenic markers overexpression by hBM-MSCs at progressively earlier time points when increasing the growth factor dose.

We elected to monitor the gene expression of TNC and SCX-A because TNC is an ECM glycoprotein considered an early marker of tenogenic differentiation, abundantly expressed in the musculoskeletal tissues during embryogenesis [65]. At the same time, SCX-A is a tendon-specific basic helix-loop-helix transcription factor responsible for the transition of MSCs into tendon progenitors [66]. Both TNC and SCX-A are particularly responsive to mechanical loading [36,37,65], though we did not study the effects of mechanical stimuli in the present study. Nevertheless, these markers were up-regulated 1.3-fold at Day 8 with 1 ng/mL of hGDF-5, suggesting an activation of the tenogenic commitment pathway after 8 days of treatment. The same activation was earlier and more pronounced when 100 ng/mL were supplemented, with SCX-A overexpressed 1.7-fold at Day 1 and TNC 1.3-fold, at Day 8. DCN was monitored because is the small leucine-rich proteoglycan involved in the regulation of fibrillogenesis, and is a fundamental component of the tendon extracellular matrix (ECM), binding to type 1 collagen fibrils [67]. This protein was again not really overexpressed by hBM-MSCs, except at Day 8 using 100 ng/mL (1.3-fold); this slight increase can be probably explained because the cells do not reach the complete organization of the ECM in all the experiments considered.

COL1A1 is the major component of tendon tissue (75–85% of the dry mass of tendon), and is responsible for its mechanical strength [68]. The up-regulation of COL1A1 gene by hBM-MSCs reached 1.5-fold, even when a dose of 100 ng/mL of GDF5 was used in our experimental setting, despite other authors reporting greater gene expression. Tan et al. [30] reported similar results to the ones evidenced in this study. Nevertheless, the expression of TNMD was significantly increased, up to 12-fold, at the concentration of 100 ng/mL of hGDF-5 after eight days of treatment, suggesting cell commitment versus the specific tendon phenotype. Indeed, TNMD is a type II transmembrane protein, commonly detected in differentiated tenocytes, and is responsible for the organization of the collagen fibers in the late phase of tendon development [2,69]. The expression of this gene was not monitored by the same authors [30], and therefore, a comparison is not possible.

Furthermore, at 100 ng/mL of hGDF-5, the COL3A1 up-regulation was reduced to only 1.3-fold after eight days; indeed, this gene is basically overexpressed by hBM-MSCs, and is the main responsible for fibrotic and scar tissue arrangement [70], and has been reported at the site of rupture of human tendons [68].

Since an early expression of SCX-A is reported to be a highly specific marker for tenocyte progenitor cell populations [36] and TNC up-regulation intervenes at the very beginning of tendon development [11], we can state that PCR data about hWJ-MSCs were favorable with a clear statistically significant overexpression of SCX-A (3.8-fold), COL1A1 (3-fold), TNC (2.3-fold) and DCN (2.7-fold) observed with 100 ng/mL of hGDF-5 at Day 3. However, hWJ-MSCs in these conditions also overexpressed COL3A1, which increased 2.8-fold, compared to the control, at Day 3. The overexpression of COL3A1 was always higher compared to hBM-MSCs. Nevertheless, we have to take into account that an early tenogenic commitment can be manifest by over-expression of SCX-A and COL1A1, an expected outcome of the tenogenic differentiation process, as stated before, because SCX-A regulates tendon formation and several other characteristics gene expression [64]. SCX-A also regulates the expression of TNMD [64], but, since TNMD is a marker of mature tenocytes, we suspect that the expression of this gene was not observed in the case of hWJ-MSCs because it would be further up-regulated if our experiments were extended to a longer period of time.

The commitment of hBM-MSCs was confirmed by quantitative immunofluorescence assay, which involved the use of Image J software Application (NHS). This investigation showed a basal level of type 1 collagen protein which was also evident in control cells, as largely documented [71], but a great overexpression of this protein was evident when 100 ng/mL were supplemented, while, tenomodulin protein was evident only at the highest hGDF-5 dose tested. Immunofluorescence (IF) observations confirmed that cells showed an ordinate aligned pattern along a specific direction at an hGDF-5 dose of 100 ng/mL at Day 16 with evident shape change in the cells, which became ovoid. The overall data are in good agreement with the literature, and confirmed that hBM-MSCs are the gold standard to set in vitro protocols to study tenogenesis or tendon differentiation and healing.

In the case of hBM-MSCs, the overall trend of type 1 collagen is in agreement with published data, suggesting that the initial phases of tenogenic commitment occurred with different timelines in MSCs, though sharing similar characteristics. Indeed, Park et al. indicated the same GDF-5 concentration as the best for ADMSCs differentiation over 12 days of cultivation [33]. The same cell populations of bone marrow MSCs were studied by Tan et al., who described similar results related to an up-regulation of the same tenogenic markers, even if related to only 10 days of hGDF-5 treatment at a concentration of 100 ng/mL [30]. In the case of hWJ-MSCs, it is difficult to put our result in another context with published data, because no similar study has been reported to date on hWJ-MSCs. However, under these experimental conditions, they seem to respond to activating tenogenic commitment events.

Regarding the mRNA levels of pro-inflammatory and anti-inflammatory cytokines, we noticed in hBM-MSCs a strong up-regulation of pro-inflammatory cytokines when the lowest concentration of hGDF-5 was used, above all IL-1β, which reached an 11.5-fold increase at Day 8. However, the strong up-regulation of IL-1β was better balanced by IL-10 (10-fold increase) when 100 ng/mL of hGDF-5 were used, suggesting that a differentiation process occurs and it could be accompanied by the immunomodulatory activity of hBM-MSCs in an anti-inflammatory fashion, as reported in the literature [58]. However, the early tenogenic commitment of hWJ-MSCs observed with 100 ng/mL at Day 3 was accompanied, similarly to hBM-MSCs, by a modulation of the immune response up-regulating the anti-inflammatory cytokine, such as IL-10 (2.5-fold). Unfortunately, to the best of our knowledge, this particular aspect of the behaviour of hWJ-MSCs have never been studied, so the hypothetic correlation between hWJ-MSCs and their immunomodulatory activity needs further investigations.

## 4. Materials and Methods

The protocol for the present study and the written informed consent were reviewed and approved by the Institutional Review Board of San Giovanni di Dio e Ruggi D’Aragona Hospital (Review Board prot./SCCE n. 24988, March 2015).

### 4.1. hBM-MSCs Isolation and Harvesting

Human Bone Marrow Mesenchymal Stem Cells (hBM-MSCs) were obtained from the bone marrow of two male donors (age 37 and 39). Donors provided written informed consent in accordance with the Declaration of Helsinki to use their filter residual bone marrow aspirate for research purposes. Briefly, total bone marrow aspirate was directly seeded at a concentration of 50,000 total nucleated cells/cm^2^ in T75 plastic flask (BD Falcon, Bedford, MA, USA) in Minimum Essential Medium Alpha (α-MEM; Corning Cellgro, Manassas, VA, USA) supplemented with 1% Glutagro^TM^ (Corning Cellgro, Manassas, VA, USA), 10% Fetal Bovine Serum (FBS; Corning Cellgro, Manassas, VA, USA), and 1% Penicillin/Streptomycin and incubated at 37 °C in 5% CO2 atmosphere and 95% relative humidity. After 72 h, non-adherent cells were removed by media change, and the adherent cells were further fed twice a week with new media. On day 14, colonies of adherent cells were detached and re-seeded at 4 × 10^3^ cells/cm^2^ in the same culture conditions. Once cultures reached 70–80% confluence, cells were detached using 0.05% trypsin-0.53mM EDTA (Corning Cellgro, Manassas, VA, USA) and washed with Phosphate-buffered saline (PBS) 1× (Corning Cellgro, Manassas, VA, USA), counted using Trypan Blue (Sigma-Aldrich, Milan, Italy) and sub-cultured at a concentration of 4 × 10^3^ cells/cm^2^. Flow Cytometry analysis was performed on cell samples obtained at Passage 1.

### 4.2. hWJ-MSCs Isolation and Harvesting

Human Wharton’s Jelly Mesenchymal Stem Cells (hWJ-MSCs) were isolated from two donors (both age 28, unrelated to the male donors) who gave written informed consent in accordance with the Declaration of Helsinki to use their umbilical cord for research purposes. hWJ-MSCs were prepared from fresh human umbilical cord obtained during normal spontaneous vaginal delivery. Briefly, umbilical cord sections, approximately 7.5 cm long, were placed in 0.9% NaCl physiological solution supplemented with Ampicillin-Sulbactam 1 g + 500 mg, stored at 4 °C, and processed within 4 h of collection. The umbilical cord was cut into 2.5 cm segments, and washed in fresh transport media to remove blood and debris. Each umbilical cord segment was sectioned longitudinally with sterile scissors, and the visible arteries and veins removed. Each piece was transferred to a tissue culture flask 175 cm^2^ (BD Falcon, Bedford, MA, USA) containing α-MEM (Corning Cellgro, Manassas, VA, USA) supplemented with 10% FBS (Corning Cellgro, Manassas, VA, USA), Glutagro^TM^ (Corning Cellgro, Manassas, VA, USA), and Penicillin-Streptomycin solution. Cultures were incubated at 37 °C in a humidified atmosphere containing 5% CO_2_. Cell growth was monitored daily with changes in media twice a week. Upon reaching 100% confluence, cells were detached using 0.05% trypsin-0.53mM EDTA (Corning Cellgro, Manassas, VA, USA) and were washed with PBS 1× (Corning Cellgro, Manassas, VA, USA), counted using Trypan Blue (Sigma-Aldrich, St. Louis, MO, USA), and sub-cultured at a concentration of 4 × 10^3^ cells/cm^2^. For hWJ-MSCs immunophenotype characterization flow cytometry analysis was performed on cell samples obtained at Passage 1.

### 4.3. Flow Cytometry

hBM-MSCs and hWJ-MSCs were detached and counted; 1 × 10^5^ cells were incubated at RT for 20 min with the following directly conjugated mouse-anti human antibodies: CD90 FITC (Beckman Coulter, Fullerton, CA, USA), CD73 APC (Miltenyi Biotec, Gladbach, Germany), CD105 PE (Beckman Coulter, Fullerton, CA, USA), CD45 PC7 (Beckman Coulter, Fullerton, CA, USA), HLA class-II FITC (Beckman Coulter, Fullerton, CA, USA), CD14 PC7 (Beckman Coulter, Fullerton, CA, USA), and CD34 PE (Beckman Coulter, Fullerton, CA, USA). The isotype-matched immunoglobulins IgG1 FITC (Beckman Coulter, Fullerton, CA, USA), IgG1 PE (Beckman Coulter, Fullerton, CA, USA), IgG1 APC (Beckman Coulter, Fullerton, CA, USA), and IgG1 PC7 (Beckman Coulter, Fullerton, CA, USA) were used as negative controls under the same conditions. At least 15,000 total events were acquired with a BD FACSVerse flow cytometer (Becton Dickinson, BD, NJ, USA). Further analysis and plots were generated using the BD FACSuite analysis software. Statistics are summarized in Table S1 (Supplementary Materials).

### 4.4. hGDF-5 Treatment

Each experiment was replicated with *n* = 3 replicates, using both individual donor cells and pooling the source. hBM-MSCs and hWJ-MSCs (passage 3) were seeded on coverslips in 12 well-plates at a concentration of 4 × 10^3^ cells/cm^2^ and 300 cells/cm^2^, respectively. The different seeding densities were to normalize for the differing cell proliferation times where seeding hWJ-MSCs at a density higher than 300 cells/cm^2^ saw them reach 90% confluence in less than three days followed by detachment from the flask surface. Once the cell cultures reached 60% confluence, cells were treated with 1 mL of culture media supplemented with three different concentrations of recombinant human GDF-5 (PeproTech; UK): 1 ng/mL, 10 ng/mL, and 100 ng/mL. Cells were fed twice a week with new media and fresh growth factor supplementation. As a consequence of the different proliferation times, hBM-MSCs were treated for 16 days, and hWJ-MSCs for seven days. Untreated cells for matched time-points were used for control purposes.

### 4.5. mRNA Isolation and Gene Expression Profile

Samples were collected at Day 1, 8 and 16 for hBM-MSCs and at Day 1, 3 and 7 for WJ-MSCs. Total mRNA was extracted using QIAzol^®^ Lysis Reagent (Qiagen, Hilden, Germany), chloroform (Sigma-Aldrich, Milan, Italy), and the RNeasy Mini Kit (Qiagen, Germany). For each sample, 1 μg of total mRNA was reverse-transcribed using the iScriptTM cDNA synthesis kit (Bio-Rad, Milan, Italy). Relative gene expression analysis was performed in a LightCycler^®^ 480 Instrument (Roche, Italy), using the SsoAdvancedTM Universal SYBR^®^ Green Supermix (Bio-Rad) with validated primers for COL1A1, COL3A1, DCN, SCX-A, TNMD, TNC, IL-6, TNF, IL-12A, IL-1β, IL-10 and TGF-β1 (Bio-Rad), and following MIQE guidelines [72]. Amplification was performed in a 10 μL final volume, including 2 ng of complementary DNA (cDNA) as a template. The specificity of the amplification products was addressed via melting curve analysis. Data were normalized to glyceraldehyde-3-phosphate dehydrogenase (GAPDH) expression (reference gene), applying the geNorm method [73] to calculate reference gene stability between the different conditions (calculated with CFX Manager software; M < 0.5). Fold changes in gene expression were determined by the 2^-ΔΔ*C*t^ method, and are presented as relative levels versus untreated cells at each time-point explored. All normalizations were obtained using untreated control samples cultivated along the investigated time points.

### 4.6. Morphometric and Proliferation Analysis

Nuclei aspect ratio of hBM-MSCs and hWJ-MSCs was determined at different time points (Day 1, 8 and 16 for hBM-MSCs; Day 1 and 7 for hWJ-MSCs) in culture by analyzing optical microscope images processed using ImageJ software. Nuclei aspect ratio was determined by measuring and dividing the length by the width of each nucleus; a total of 50–80 nuclei was measured in each condition using a minimum of 10 microscope images.

Proliferation of hBM-MSCs and hWJ-MSCs was quantified by counting the number of nuclei in each time point (Day 0 and 16 for hBM-MSCs; Day 0 and 7 for hWJ-MSCs) and in each condition of treatment (1 ng/mL, 10 ng/mL and 100 ng/mL of hGDF-5) using a minimum of 10 microscope images.

### 4.7. Immunofluorescence Assay

Samples were collected at Day 1, 8 and 16 for hBM-MSCs and at Day 1 and 7 for WJ-MSCs. Cells were fixed with 3.7% formaldehyde for 30 min at room temperature (RT) followed by permeabilization with 0.1% Triton X-100 for 5 min and blocking with 1% BSA for 1 h. For type 1 collagen and tenomodulin staining, cells were incubated overnight at 4 °C with a mouse monoclonal anti-type 1 collagen antibody (1:100, Sigma-Aldrich, Milan, Italy) and a rabbit polyclonal anti-tenomodulin antibody (1:100; Abcam, Cambridge, United Kingdom). Following incubation with the primary antibody, cells were incubated for 1 h at RT with the DyLight 649 anti-mouse IgG (1:500, BioLegend, CA, USA) antibody and the Alexa FluorTM 488 goat anti-rabbit IgG (1:500; Thermo Fisher Scientific, Waltham, MA, USA). Cell nuclei were stained with DAPI solution (1:1000) for 5 min. Images acquisition was at 63× magnification using an inverted Leica laser-scanning confocal microscope (TCS SP5; Leica Microsystems, Wetzlar, Germany) equipped with a plan Apo 63x/1.4 NA oil immersion objective.

Image quantification was performed in a blinded manner using image analysis software (ImageJ, National Institutes of Health, Bethesda, MD, USA) by measuring the red and green areas where type 1 collagen and tenomodulin, respectively, are expressed [74,75]. Original images were converted from RGB format into a gray scale (16-bit). Then, the average value of pixel intensity (within a range from 0-dark to 255-white) was calculated for each image. Signal intensity was normalized by the cell number (e.g., by the amount of cell nuclei revealed by DAPI staining). A minimum of 10 image fields (with a comparable number of cells in each image) were used for the analysis at each time point for each experiment.

Data were expressed as fold change relative to untreated cells at matched time-points.

### 4.8. Statistical Analysis

Statistical analysis was performed using GraphPad Prism software (6.0 for Windows). For phenotypic analysis, data obtained from multiple experiments (*n* = 3) are calculated as mean+/-SD and analyzed for statistical significance using ANOVA test, for independent groups. Differences were considered statistically significant when *p* ≤ 0.05.

## 5. Conclusions

Although the use of autologous stem cells for tendon healing and regeneration has been described and all tissue-engineering studies indicated bone marrow stem cells as the gold standard to promote tendon regeneration, hWJ-MSCs have never been studied in this sense and may be an interesting option. Furthermore, a better understanding of the complex interactions and pathways of the biomolecules involved in targeted tissue regeneration is necessary to achieve effective therapeutic outcomes for translation into clinic practice. In this context, hGDF-5 induces cellular events of tenogenic differentiation that can be time and concentration-dependent. The concentration of 100 ng/mL is more effective in this sense, correlating with an anti-inflammatory modulation of the response of the immune cells. This behavior was observed on both stem cells, bone marrow and umbilical cord derived. While the proliferation of hBM-MSCs is only slightly promoted during hGDF-5 treatment, the hWJ-MSCs population maintained a high proliferation rate. After seven days and at the dose of 100 ng/mL of hGDF-5, hWJ-MSCs manifested the up-regulation of tenogenic markers and showed L/W ratio > 1.7. These preliminary data suggested that, given their properties, hWJ-MSCs could be potentially used in in vitro tendon tissue-engineering. Further questions, such as the role of proteoglycans expression in the modulation of tenogenic commitment in combination with GDF-5 signaling and the nature of epigenetic signature of both cell types have also to be investigated in the future to further understand the different proliferative rate and markers expression.

## Supplementary Materials

The following are available online at https://www.mdpi.com/1422-0067/21/16/5905/s1. Table S1: Flow cytometry data, Figure S1: Brightfield images of hBM-MSCs and hWJ-MSCs with the hGDF-5 dose-dependent effect, Figure S2: Immunofluorescence images (split channels) illustrating the effect of hGDF-5 treatment on the expression of type 1 collagen and tenomodulin proteins on hBM-MSCs, Figure S3: Immunofluorescence images (split channels) illustrating the effect of hGDF-5 treatment on the expression of type 1 collagen and tenomodulin proteins on hWJ-MSCs.

## Abbreviations

hGDF-5	human Growth Differentiation Factor-5
hMSCs	human Mesenchymal Stem Cells
hBM-MSCs	human Bone Marrow Mesenchymal Stem Cells
hWJ-MSCs	human Wharton’s Jelly Mesenchymal Stem Cells
COL1A1	type 1 collagen
COL3A1	type 3 collagen
DCN	Decorin
SCX-A	Scleraxis A
TNC	Tenascin-C
TNMD	Tenomodulin
RT-PCR	Real-Time Polymerase Chain Reaction
IF	Immunofluorescence
q-IF	Quantitative Immunofluorescence assay with ImageJ software (NHS)

## References

[1] Gaspar D., Spanoudes K., Holladay C., Pandit A., Zeugolis D. (2015). Progress in cell-based therapies for tendon repair. Adv. Drug Deliv. Rev..

[2] Docheva D., Müller S.A., Majewski M., Evans C.H. (2015). Biologics for tendon repair. Adv. Drug Deliv. Rev..

[3] Burk J. (2019). Mechanisms of Action of Multipotent Mesenchymal Stromal Cells in Tendon Disease. Tendons.

[4] Sharma P., Maffulli N. (2006). Biology of tendon injury: Healing, modeling and remodeling. J. Musculoskelet. Neuronal Interact..

[5] Giordano L., Della Porta G., Peretti G.M., Maffulli N. (2020). Therapeutic potential of microRNA in tendon injuries. Br. Med. Bull..

[6] Dakin S.G., Dudhia J., Smith R.K.W. (2013). Science in brief: Resolving tendon inflammation. A new perspective: Resolving tendon inflammation. Equine Vet. J..

[7] Derwin K.A., Baker A.R., Spragg R.K., Leigh D.R., Iannotti J.P. (2006). Commercial Extracellular Matrix Scaffolds for Rotator Cuff Tendon Repair: Biomechanical, Biochemical, and Cellular Properties. J. Bone Jt. Surg..

[8] Chen J., Xu J., Wang A., Zheng M. (2009). Scaffolds for tendon and ligament repair: Review of the efficacy of commercial products. Expert Rev. Med. Devices.

[9] Smith R.K.W., Werling N.J., Dakin S.G., Alam R., Goodship A.E., Dudhia J. (2013). Beneficial Effects of Autologous Bone Marrow-Derived Mesenchymal Stem Cells in Naturally Occurring Tendinopathy. PLoS ONE.

[10] Pak J., Lee J.H., Park K.S., Park M., Kang L.-W., Lee S.H. (2017). Current use of autologous adipose tissue-derived stromal vascular fraction cells for orthopedic applications. J. Biomed. Sci..

[11] Bourin P., Bunnell B.A., Casteilla L., Dominici M., Katz A.J., March K.L., Redl H., Rubin J.P., Yoshimura K., Gimble J.M. (2013). Stromal cells from the adipose tissue-derived stromal vascular fraction and culture expanded adipose tissue-derived stromal/stem cells: A joint statement of the International Federation for Adipose Therapeutics and Science (IFATS) and the International Society for Cellular Therapy (ISCT). Cytotherapy.

[12] Migliorini F., Tingart M., Maffulli N. (2020). Progress with stem cell therapies for tendon tissue regeneration. Expert Opin. Biol. Ther..

[13] Andia I., Maffulli N. (2019). New biotechnologies for musculoskeletal injuries. Surgeon.

[14] Veronesi F., Salamanna F., Tschon M., Maglio M., Nicoli Aldini N., Fini M. (2017). Mesenchymal stem cells for tendon healing: What is on the horizon?: Mesenchymal stem cells in acute and chronic tendon injuries. J. Tissue Eng. Regen. Med..

[15] Costa-Almeida R., Calejo I., Gomes M.E. (2019). Mesenchymal Stem Cells Empowering Tendon Regenerative Therapies. Int. J. Mol. Sci..

[16] Sharma P., Maffulli N. (2005). Tendon Injury and Tendinopathy: Healing and Repair. J. Bone Jt. Surg..

[17] Gonçalves A.I., Rodrigues M.T., Lee S.-J., Atala A., Yoo J.J., Reis R.L., Gomes M.E. (2013). Understanding the Role of Growth Factors in Modulating Stem Cell Tenogenesis. PLoS ONE.

[18] Lui P.P.Y., Rui Y.F., Ni M., Chan K.M. (2011). Tenogenic differentiation of stem cells for tendon repair-what is the current evidence?. J. Tissue Eng. Regen. Med..

[19] Caplan A.I., Bruder S.P. (2001). Mesenchymal stem cells: Building blocks for molecular medicine in the 21st century. Trends Mol. Med..

[20] Dominici M., Le Blanc K., Mueller I., Slaper-Cortenbach I., Marini F.C., Krause D.S., Deans R.J., Keating A., Prockop D.J., Horwitz E.M. (2006). Minimal criteria for defining multipotent mesenchymal stromal cells. The International Society for Cellular Therapy position statement. Cytotherapy.

[21] Hankemeier S., Keus M., Zeichen J., Jagodzinski M., Barkhausen T., Bosch U., Krettek C., Griensven M.V. (2005). Modulation of Proliferation and Differentiation of Human Bone Marrow Stromal Cells by Fibroblast Growth Factor 2: Potential Implications for Tissue Engineering of Tendons and Ligaments. Tissue Eng..

[22] Wang D., Jiang X., Lu A., Tu M., Huang W., Huang P. (2018). BMP14 induces tenogenic differentiation of bone marrow mesenchymal stem cells in vitro. Exp. Ther. Med..

[23] Citeroni M.R., Ciardulli M.C., Russo V., Della Porta G., Mauro A., El Khatib M., Di Mattia M., Forsyth N.R., Galesso D., Barbera C. (2020). In vitro innovation of tendon tissue engineering strategies. Int. J. Mol. Sci..

[24] Chang S.C., Hoang B., Thomas J.T., Vukicevic S., Luyten F.P., Ryba N.J., Kozak C.A., Reddi A.H., Moos M. (1994). Cartilage-derived morphogenetic proteins. New members of the transforming growth factor-beta superfamily predominantly expressed in long bones during human embryonic development. J. Biol. Chem..

[25] Zhou S., Yates K.E., Eid K., Glowacki J. (2005). Demineralized bone promotes chondrocyte or osteoblast differentiation of human marrow stromal cells cultured in collagen sponges. Cell Tissue Bank..

[26] Wolfman N.M., Hattersley G., Cox K., Celeste A.J., Nelson R., Yamaji N., Dube J.L., DiBlasio-Smith E., Nove J., Song J.J. (1997). Ectopic induction of tendon and ligament in rats by growth and differentiation factors 5, 6, and 7, members of the TGF-beta gene family. J. Clin. Investig..

[27] Forslund C., Rueger D., Aspenberg P. (2003). A comparative dose–response study of cartilage-derived morphogenetic protein (CDMP)-1, -2 and -3 for tendon healing in rats. J. Orthop. Res..

[28] Keller T.C., Hogan M.V., Kesturu G., James R., Balian G., Chhabra A.B. (2011). Growth/differentiation factor-5 modulates the synthesis and expression of extracellular matrix and cell-adhesion-related molecules of rat Achilles tendon fibroblasts. Connect. Tissue Res..

[29] Hogan M., Girish K., James R., Balian G., Hurwitz S., Chhabra A.B. (2011). Growth differentiation factor-5 regulation of extracellular matrix gene expression in murine tendon fibroblasts. J. Tissue Eng. Regen. Med..

[30] Tan S.-L., Ahmad R.E., Ahmad T.S., Merican A.M., Abbas A.A., Ng W.M., Kamarul T. (2012). Effect of Growth Differentiation Factor 5 on the Proliferation and Tenogenic Differentiation Potential of Human Mesenchymal Stem Cells in vitro. Cells Tissues Organs.

[31] Ozasa Y., Gingery A., Thoreson A.R., An K.-N., Zhao C., Amadio P.C. (2014). A Comparative Study of the Effects of Growth and Differentiation Factor 5 on Muscle-Derived Stem Cells and Bone Marrow Stromal Cells in an In Vitro Tendon Healing Model. J. Hand Surg..

[32] Shwartz Y., Viukov S., Krief S., Zelzer E. (2016). Joint Development Involves a Continuous Influx of Gdf5-Positive Cells. Cell Rep..

[33] Park A., Hogan M.V., Kesturu G.S., James R., Balian G., Chhabra A.B. (2010). Adipose-Derived Mesenchymal Stem Cells Treated with Growth Differentiation Factor-5 Express Tendon-Specific Markers. Tissue Eng. Part A.

[34] Bottagisio M., Lopa S., Granata V., Talò G., Bazzocchi C., Moretti M., Barbara Lovati A. (2017). Different combinations of growth factors for the tenogenic differentiation of bone marrow mesenchymal stem cells in monolayer culture and in fibrin-based three-dimensional constructs. Differentiation.

[35] Tan S.-L., Ahmad T.S., Ng W.-M., Azlina A.A., Azhar M.M., Selvaratnam L., Kamarul T. (2015). Identification of Pathways Mediating Growth Differentiation Factor5-Induced Tenogenic Differentiation in Human Bone Marrow Stromal Cells. PLoS ONE.

[36] Govoni M., Berardi A.C., Muscari C., Campardelli R., Bonafè F., Guarnieri C., Reverchon E., Giordano E., Maffulli N., Della Porta G. (2017). An Engineered Multiphase Three-Dimensional Microenvironment to Ensure the Controlled Delivery of Cyclic Strain and Human Growth Differentiation Factor 5 for the Tenogenic Commitment of Human Bone Marrow Mesenchymal Stem Cells. Tissue Eng. Part A.

[37] Ciardulli M.C., Marino L., Lovecchio J., Giordano E., Forsyth N.R., Selleri C., Maffulli N., Della Porta G. (2020). Tendon and Cytokine Marker Expression by Human Bone Marrow Mesenchymal Stem Cells in a Hyaluronate/Poly-Lactic-Co-Glycolic Acid (PLGA)/Fibrin Three-Dimensional (3D) Scaffold. Cells.

[38] Rinoldi C., Fallahi A., Yazdi I.K., Campos Paras J., Kijeńska-Gawrońska E., Trujillo-de Santiago G., Tuoheti A., Demarchi D., Annabi N., Khademhosseini A. (2019). Mechanical and Biochemical Stimulation of 3D Multilayered Scaffolds for Tendon Tissue Engineering. ACS Biomater. Sci. Eng..

[39] Baksh D., Yao R., Tuan R.S. (2007). Comparison of Proliferative and Multilineage Differentiation Potential of Human Mesenchymal Stem Cells Derived from Umbilical Cord and Bone Marrow. Stem Cells.

[40] Wang H.-S., Hung S.-C., Peng S.-T., Huang C.-C., Wei H.-M., Guo Y.-J., Fu Y.-S., Lai M.-C., Chen C.-C. (2004). Mesenchymal Stem Cells in the Wharton’s Jelly of the Human Umbilical Cord. Stem Cells.

[41] Marino L., Castaldi M.A., Rosamilio R., Ragni E., Vitolo R., Fulgione C., Castaldi S.G., Serio B., Bianco R., Guida M. (2019). Mesenchymal Stem Cells from the Wharton’s Jelly of the Human Umbilical Cord: Biological Properties and Therapeutic Potential. Int. J. Stem Cells.

[42] Ding D.-C., Chang Y.-H., Shyu W.-C., Lin S.-Z. (2015). Human Umbilical Cord Mesenchymal Stem Cells: A New Era for Stem Cell Therapy. Cell Transplant..

[43] Fong C.-Y., Chak L.-L., Biswas A., Tan J.-H., Gauthaman K., Chan W.-K., Bongso A. (2011). Human Wharton’s Jelly Stem Cells Have Unique Transcriptome Profiles Compared to Human Embryonic Stem Cells and Other Mesenchymal Stem Cells. Stem Cell Rev. Rep..

[44] Kuroda Y. (2011). Mesenchymal Stem Cells and Umbilical Cord as Sources for Schwann Cell Differentiation: Their Potential in Peripheral Nerve Repair. Open Tissue Eng. Regen. Med. J..

[45] Du T., Zou X., Cheng J., Wu S., Zhong L., Ju G., Zhu J., Liu G., Zhu Y., Xia S. (2013). Human Wharton’s jelly-derived mesenchymal stromal cells reduce renal fibrosis through induction of native and foreign hepatocyte growth factor synthesis in injured tubular epithelial cells. Stem Cell Res. Ther..

[46] Moodley Y., Atienza D., Manuelpillai U., Samuel C.S., Tchongue J., Ilancheran S., Boyd R., Trounson A. (2009). Human Umbilical Cord Mesenchymal Stem Cells Reduce Fibrosis of Bleomycin-Induced Lung Injury. Am. J. Pathol..

[47] Lo Iacono M. (2011). Perinatal and Wharton’s Jelly-Derived Mesenchymal Stem Cells in Cartilage Regenerative Medicine and Tissue Engineering Strategies. Open Tissue Eng. Regen. Med. J..

[48] Scheers I. (2011). Cell Therapy for the Treatment of Metabolic Liver Disease: An Update on the Umbilical Cord Derived Stem Cells Candidates. Open Tissue Eng. Regen. Med. J..

[49] Tamura M. (2011). Wharton’s Jelly Stem Cells as Agents for Cancer Therapy. Open Tissue Eng. Regen. Med. J..

[50] Karahuseyinoglu S., Cinar O., Kilic E., Kara F., Akay G.G., Demiralp D.Ö., Tukun A., Uckan D., Can A. (2007). Biology of Stem Cells in Human Umbilical Cord Stroma: In Situ and In Vitro Surveys. Stem Cells.

[51] Sarugaser R., Lickorish D., Baksh D., Hosseini M.M., Davies J.E. (2005). Human Umbilical Cord Perivascular (HUCPV) Cells: A Source of Mesenchymal Progenitors. Stem Cells.

[52] Fu Y.-S., Shih Y.-T., Cheng Y.-C., Min M.-Y. (2004). Transformation of human umbilical mesenchymal cells into neurons in vitro. J. Biomed. Sci..

[53] Conconi M.T., Burra P., Di Liddo R., Calore C., Turetta M., Bellini S., Bo P., Nussdorfer G.G., Parnigotto P.P. (2006). CD105(+) cells from Wharton’s jelly show in vitro and in vivo myogenic differentiative potential. Int. J. Mol. Med..

[54] Yea J.-H., Bae T.S., Kim B.J., Cho Y.W., Jo C.H. (2020). Regeneration of the rotator cuff tendon-to-bone interface using umbilical cord-derived mesenchymal stem cells and gradient extracellular matrix scaffolds from adipose tissue in a rat model. Acta Biomater..

[55] Rak Kwon D., Jung S., Jang J., Park G.-Y., Suk Moon Y., Lee S.C. (2020). A 3-Dimensional Bioprinted Scaffold With Human Umbilical Cord Blood–Mesenchymal Stem Cells Improves Regeneration of Chronic Full-Thickness Rotator Cuff Tear in a Rabbit Model. Am. J. Sports Med..

[56] Sugg K.B., Lubardic J., Gumucio J.P., Mendias C.L. (2014). Changes in macrophage phenotype and induction of epithelial-to-mesenchymal transition genes following acute Achilles tenotomy and repair: Tendon Macrophage Phenotype and Emt. J. Orthop. Res..

[57] Gao F., Chiu S.M., Motan D.A.L., Zhang Z., Chen L., Ji H.-L., Tse H.-F., Fu Q.-L., Lian Q. (2016). Mesenchymal stem cells and immunomodulation: Current status and future prospects. Cell Death Dis..

[58] Kim D., Yoo K., Choi K., Choi J., Choi S., Yang S., Yang Y., Im H., Kim K., Jung H. (2005). Gene expression profile of cytokine and growth factor during differentiation of bone marrow-derived mesenchymal stem cell. Cytokine.

[59] Gruber H.E., Hoelscher G.L., Ingram J.A., Bethea S., Hanley E.N. (2014). Growth and differentiation factor-5 (GDF-5) in the human intervertebral annulus cells and its modulation by IL-1ß and TNF-α in vitro. Exp. Mol. Pathol..

[60] Mabuchi Y., Houlihan D.D., Akazawa C., Okano H., Matsuzaki Y. (2013). Prospective Isolation of Murine and Human Bone Marrow Mesenchymal Stem Cells Based on Surface Markers. Stem Cells Int..

[61] El Khatib M., Mauro A., Di Mattia M., Wyrwa R., Schweder M., Ancora M., Lazzaro F., Berardinelli P., Valbonetti L., Di Giacinto O. (2020). Electrospun PLGA Fiber Diameter and Alignment of Tendon Biomimetic Fleece Potentiate Tenogenic Differentiation and Immunomodulatory Function of Amniotic Epithelial Stem Cells. Cells.

[62] Chuen F.S., Chuk C.Y., Ping W.Y., Nar W.W., Kim H.L., Ming C.K. (2004). Immunohistochemical Characterization of Cells in Adult Human Patellar Tendons. J. Histochem. Cytochem..

[63] Barboni B., Curini V., Russo V., Mauro A., Di Giacinto O., Marchisio M., Alfonsi M., Mattioli M. (2012). Indirect Co-Culture with Tendons or Tenocytes Can Program Amniotic Epithelial Cells towards Stepwise Tenogenic Differentiation. PLoS ONE.

[64] Donders R., Bogie J.F.J., Ravanidis S., Gervois P., Vanheusden M., Marée R., Schrynemackers M., Smeets H.J.M., Pinxteren J., Gijbels K. (2018). Human Wharton’s Jelly-Derived Stem Cells Display a Distinct Immunomodulatory and Proregenerative Transcriptional Signature Compared to Bone Marrow-Derived Stem Cells. Stem Cells Dev..

[65] Järvinen T.A., Jozsa L., Kannus P., Järvinen T.L., Kvist M., Hurme T., Isola J., Kalimo H., Järvinen M. (1999). Mechanical loading regulates tenascin-C expression in the osteotendinous junction. J. Cell Sci..

[66] Alberton P., Popov C., Prägert M., Kohler J., Shukunami C., Schieker M., Docheva D. (2012). Conversion of Human Bone Marrow-Derived Mesenchymal Stem Cells into Tendon Progenitor Cells by Ectopic Expression of Scleraxis. Stem Cells Dev..

[67] Wagenhäuser M.U., Pietschmann M.F., Sievers B., Docheva D., Schieker M., Jansson V., Müller P.E. (2012). Collagen type I and decorin expression in tenocytes depend on the cell isolation method. BMC Musculoskelet. Disord..

[68] Pajala A., Melkko J., Leppilahti J., Ohtonen P., Soini Y., Risteli J. (2009). Tenascin-C and type I and III collagen expression in total Achilles tendon rupture. An immunohistochemical study. Histol. Histopathol..

[69] Tokunaga T., Shukunami C., Okamoto N., Taniwaki T., Oka K., Sakamoto H., Ide J., Mizuta H., Hiraki Y. (2015). FGF-2 Stimulates the Growth of Tenogenic Progenitor Cells to Facilitate the Generation of *Tenomodulin* -Positive Tenocytes in a Rat Rotator Cuff Healing Model. Am. J. Sports Med..

[70] Williams I.F., Heaton A., McCullagh K.G. (1980). Cell morphology and collagen types in equine tendon scar. Res. Vet. Sci..

[71] Jo C.H., Lim H.-J., Yoon K.S. (2019). Characterization of Tendon-Specific Markers in Various Human Tissues, Tenocytes and Mesenchymal Stem Cells. Tissue Eng. Regen. Med..

[72] Bustin S.A., Benes V., Garson J.A., Hellemans J., Huggett J., Kubista M., Mueller R., Nolan T., Pfaffl M.W., Shipley G.L. (2009). The MIQE Guidelines: Minimum Information for Publication of Quantitative Real-Time PCR Experiments. Clin. Chem..

[73] Hellemans J., Mortier G., De Paepe A., Speleman F., Vandesompele J. (2007). qBase relative quantification framework and software for management and automated analysis of real-time quantitative PCR data. Genome Biol..

[74] Jensen E.C. (2013). Quantitative Analysis of Histological Staining and Fluorescence Using ImageJ: Histological Staining/Fluorescence Using ImageJ. Anat. Rec..

[75] Rinoldi C., Costantini M., Kijeńska-Gawrońska E., Testa S., Fornetti E., Heljak M., Ćwiklińska M., Buda R., Baldi J., Cannata S. (2019). Tendon Tissue Engineering: Effects of Mechanical and Biochemical Stimulation on Stem Cell Alignment on Cell-Laden Hydrogel Yarns. Adv. Healthc. Mater..

