# Supplementary material for: Dose-Response Tendon-Specific Markers Induction by Growth Differentiation Factor-5 in Human Bone Marrow and Umbilical Cord Mesenchymal Stem Cells
Source: Int J Mol Sci. 2020 Aug 17;21(16):5905. doi: 10.3390/ijms21165905 (PMC7460605; doi:10.3390/ijms21165905)
Supplement: Supplementary file 1 [file ijms-21-05905-s001.pdf]

Table S1. Flow cytometry statistics data of all the main events considered for the population of hBM-MSCs and hWJ-MSCs used in the experiments. All samples were positive for CD90, CD105, CD73 and negative for CD14, CD34, CD45, HLA-DR.

Sample Name	# of Total Events	% Parent (Gated on P1)	% Total (No Gate)
**hBM-MSC all events**	15,000	***	100,00
hBM-MSC: P1	13,364	89.09	89.09
hBM-MSC: HLA-DR+	72	0.54	0.48
hBM-MSC:CD34+	87	0.65	0.58
hBM-MSC:CD14+	258	1.93	1.72
**hBM-MSC all events**	15,000	***	100.00
hBM-MSC: P1	13,555	90.37	90.37
hBM-MSC:CD90+	13,494	99.55	89.96
hBM-MSC:CD105+	13,538	99.87	90.25
hBM-MSC:CD73+	13,537	99.87	90.25
hBM-MSC:CD45+	35	0.26	0.23
**Sample Name**	**# of Total Events**	**% Parent (Gated on P1)**	**% Total (No Gate)**
**hWJ-MSC all events**	15,000	***	100.00
hWJ -MSC: P1	11,619	77.46	77.46
hWJ -MSC: HLA-DR+	41	0.35	0.27
hWJ -MSC:CD34+	31	0.27	0.21
hWJ -MSC:CD14+	21	0.18	0.14
**hWJ -MSC all events**	15,000	***	100.00
hWJ -MSC: P1	12,465	83.10	83.10
hWJ -MSC:CD90+	12,368	99.22	82.45
hWJ -MSC:CD105+	12,379	99.31	82.53
hWJ -MSC:CD73+	12,369	99.23	82.46
hWJ -MSC:CD45+	104	0.83	0.69

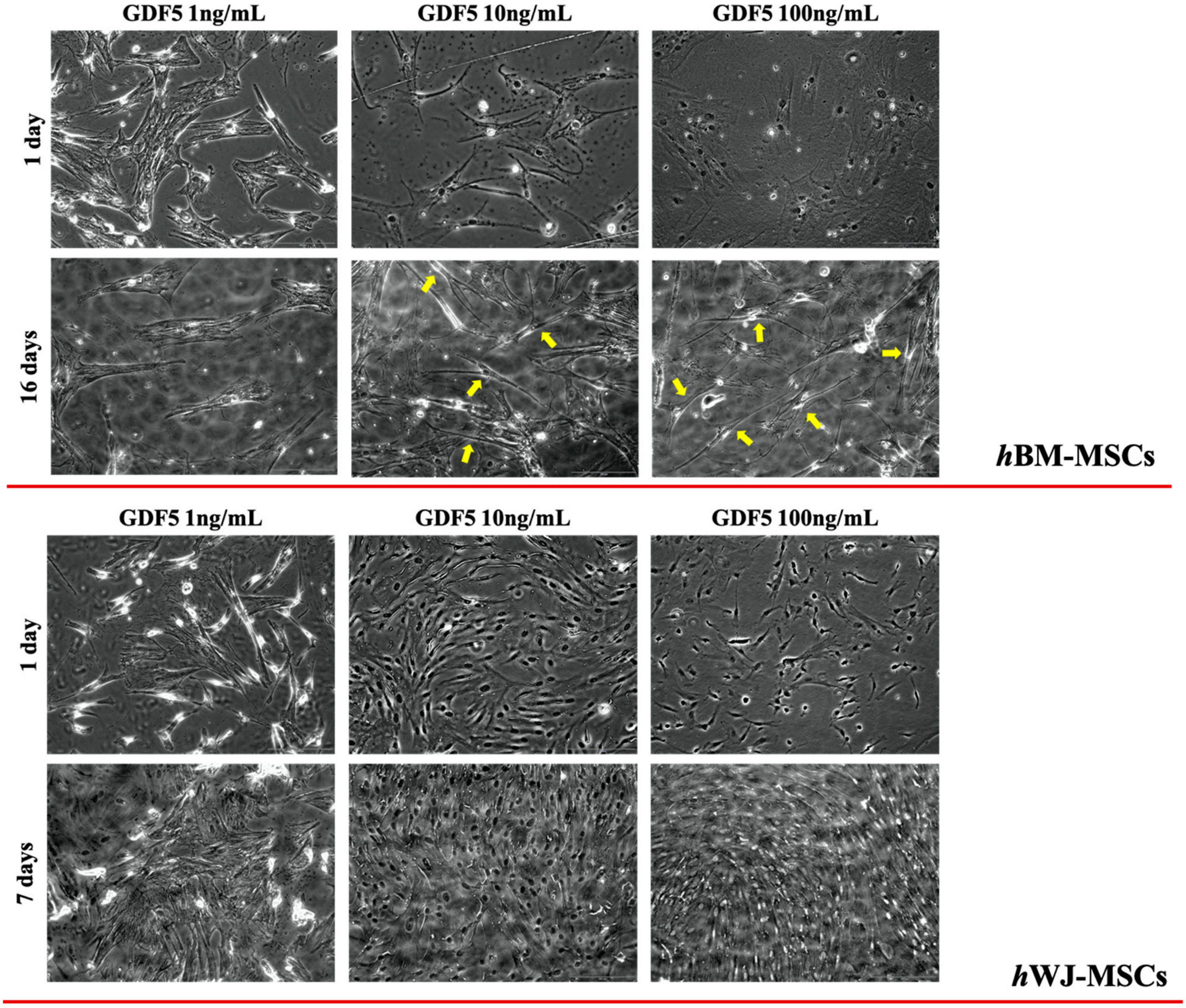

Figure S1. Brightfield images of hBM-MSCs and hWJ-MSCs with the hGDF-5 dose-dependent effect. Both cells showed cells specific alignment and their shape modification; hWJ-MSCs exhibited always higher proliferation rate positively affected by 100 ng/mL of GDF-5 dose.

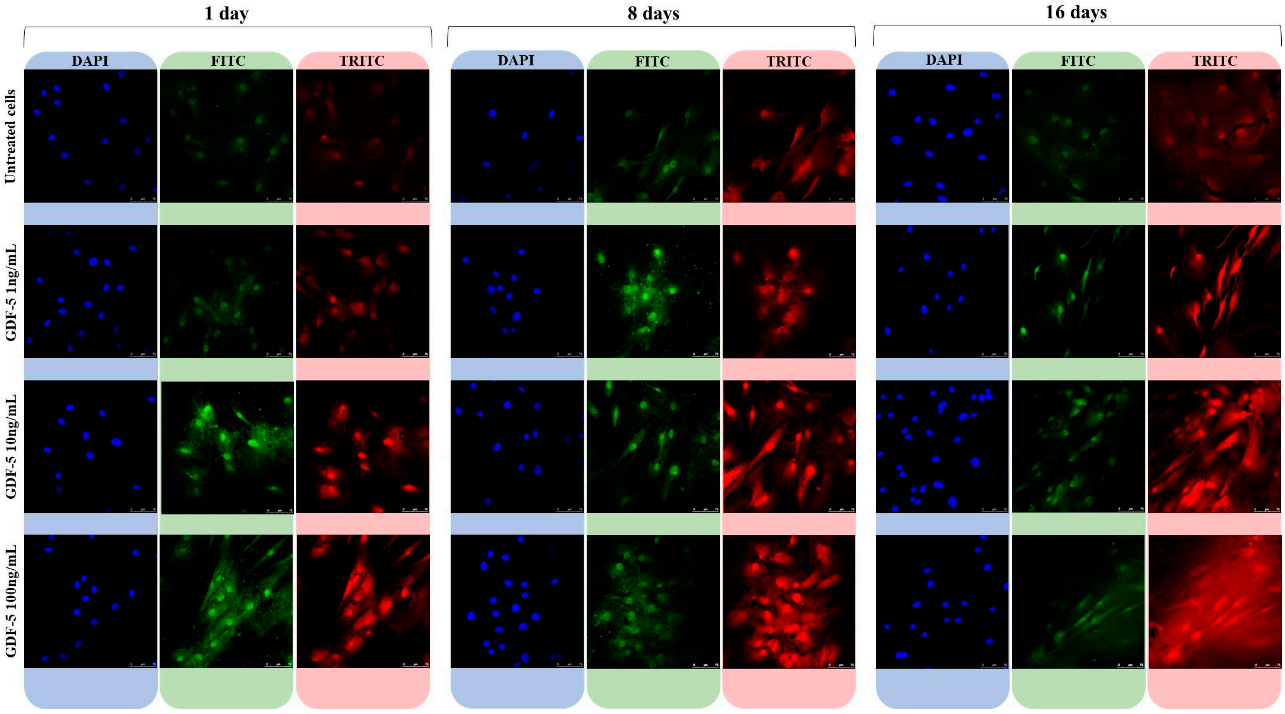

Figure S2. Immunofluorescence images illustrating the effect of hGDF-5 dose on the expression of type 1 collagen and tenomodulin proteins on hBM-MSCs up to 16 days of culture. The panel shows the split color channels: type 1 collagen (red staining) and tenomodulin (green staining).

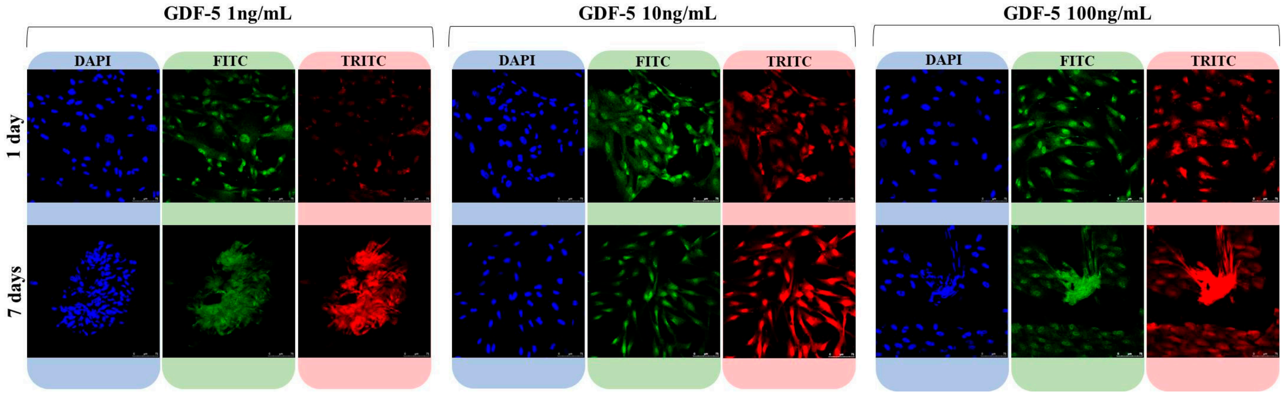

Figure S3. Immunofluorescence images illustrating the effect of hGDF-5 dose on the expression of type I collagen and tenomodulin proteins on hWJ-MSCs up to seven days of culture. The panel shows the split color channels: type 1 collagen (red staining) and tenomodulin (green staining).
